# Hepatitis B virus inhibits apolipoprotein A5 expression through its core gene

**DOI:** 10.1186/s12944-016-0340-2

**Published:** 2016-10-10

**Authors:** Chengliang Zhu, Guosheng Gao, Hui Song, Fengxia Xu, Kailang Wu, Xinghui Liu

**Affiliations:** 1 Department of Clinical Laboratory, Renmin Hospital of Wuhan University, Wuhan, Hubei 430060 People’s Republic of China; 2 Department of Clinical Laboratory, Ningbo NO. 2 Hospital, Ningbo, 315010 People’s Republic of China; 3 Department of Clinical Laboratory, Gongli Hospital, Second Military Medicine University, Pudong New Area, Shanghai, 200135 People’s Republic of China; 4 The State Key Laboratory of Virology, College of Life Sciences, Wuhan University, Wuhan, Hubei 430072 People’s Republic of China

**Keywords:** Hepatitis B virus, Apolipoprotein A5, Expression

## Abstract

**Background:**

Hepatitis B virus (HBV) infection causes lipid metabolism disorders. Apolipoprotein A5 (ApoA5) is a new apolipoprotein family member that plays an important role in the regulation of lipid metabolism. The present study was to investigate the impact of HBV on ApoA5 expression and its regulatory mechanism.

**Methods:**

Reverse transcription polymerase chain reaction (RT-PCR) and western blotting were used to measure ApoA5 mRNA and protein expression in HepG2 and HepG2.2.15 cells. Enzyme-linked immunosorbent assay (ELISA) was used to measure the serum ApoA5 levels in healthy individuals and HBV patients. HBV infectious clone pHBV1.3 or individual plasmids expressing the HBV genome was cotransfected with the ApoA5 promoter pGL3-Apo5-LUC plasmid into HepG2 cells to assess the luciferase activity. RT-PCR and western blotting methods were used to detect Apo5 mRNA and protein expression, respectively.

**Results:**

The ApoA5 mRNA and protein expression levels were decreased in HepG2.2.15 cells compared with the control HepG2 cells. The serum ApoA5 levels were 196.4 ± 28.7 μg/L in the healthy individuals and 104.5 ± 18.3 μg/L in the HBV patients, statistical analysis showed that the ApoA5 levels were significantly lower in HBV patients than in the healthy individuals (*P* < 0.05). pHBV1.3 and its core gene inhibited ApoA5 promoter activity and mRNA and protein expression in HepG2 cells.

**Conclusion:**

HBV inhibits ApoA5 expression at both the transcriptional and translational levels through its core gene.

## Background

Hepatitis B virus (HBV) is a DNA virus belonging to *Hepadnaviridae* with a viral genome containing approximately 3200 base pairs. To date, there are approximately 350 million HBV carriers around the globe, and up to 50 million people are infected with HBV each year [1, 2]. The HBV genome contains approximately 3200 base pairs and 4 open reading frames (S/PreS, C/PreC, P and X). S/PreS encodes 3 surface proteins (PreS1, PreS2 and S), C/PreC encodes the signal peptide and core protein (PreC), P encodes the DNA polymerase (P), and X encodes the X protein (HBx) [3].

Apolipoprotein A5 (ApoA5) is a new member of the apolipoprotein family and is specifically synthesized and secreted by the liver. ApoA5 is present in high-density lipoprotein (HDL), very low-density lipoprotein (VLDL) and chylomicrons (CMs) but is not present in other plasma lipoproteins [4]. Studies have shown that HBV infection can cause blood lipid metabolism disorder [5]. However, there have been no reports concerning the relationship between HBV and ApoA5. The present study investigated the impact of HBV on ApoA5 expression and explored its regulatory mechanism.

## Methods

### Study subjects

We collected 221 cases of clinically diagnosed HBV patients with an average age of 51.6 ± 11.8 years, of whom 120 were males and 101 were females. None of the patients had diseases of the heart, brain, kidney or other important organs, other chronic liver diseases, or diseases that could cause metabolic disorders. A total of 125 healthy individuals with an average age of 48.7 ± 13.6 years were used as the normal control group, of whom 75 were males and 50 were females. The work was approved by the Ethical Committee and written informed consents were obtained from all participating individuals.

### Cell culture and transfection

HepG2 and HepG2.2.15 cells were cultured in RPMI 1640 medium containing 10 % foetal bovine serum, 100 U/mL of penicillin and 100 mg/L of streptomycin in a 5 % CO2 and 37 °C incubator. Prior to transfection, the HepG2 cells were seeded into 24-well or 6-well plates. Cell transfection was performed according to the following procedure. Plasmid DNA and 2 μL of Lipofectamine 2000 (Invitrogen, U.S.A) were diluted in 30 μL of RPMI-1640, or 4 μg of plasmid DNA and 6 μL of Lipofectamine 2000 were diluted in 100 μL of RPMI-1640. The mixtures were incubated at room temperature for 20 min. Then, the prepared transfection mixture was added to the cell culture medium in the 24-well or 6-well plates. The cells were cultured in a CO2 incubator. The transfection efficiency of HepG2 cells was evaluated by transfected with pIRES2-EGFP.

### Reverse Transcriptase (RT)-PCR detection

TRIzol R (Invitrogen, Carlsbad, CA, USA) was used to isolate total cellular RNA. Reverse transcription was performed to synthesize cDNA for use as a template. For ApoA5 gene detection, the sense primer 5′ TGGGCTCTGGCTCTTCTTT 3’ and the antisense primer 5′ ACCTCCTCCAACTCCTCCTG 3’ were used for PCR amplification. β-actin was used an internal control. The product was verified by 1 % agarose gel electrophoresis.

### Measurement of luciferase activity

The transfected cells were cultured for 48 h. Then, the cells were harvested, lysis buffer was added to lyse the cells, and 10 μL of the cell lysate was mixed with 100 μL of the luciferase substrate. A luminometer was used to measure the luciferase activities [6].

### Western blotting analysis

Transfected HepG2 cells were collected and lysed in lysis buffer using sonication. The lysates were centrifuged to collect the proteins in the supernatant. A 20 mg protein sample was mixed with an equal volume of sample buffer. The mixture was boiled in water for 5 min and loaded onto a 12 % SDS-PAGE gel for separation. After electrophoresis, the proteins were transferred onto a nitrocellulose (NC) membrane. The membrane was blocked with 5 % fat-free milk dissolved in phosphate-buffered saline plus Tween 20 (PBST) for 2 h. ApoA5 monoclonal antibody (Santa Cruz Biotechnology Inc., Santa Cruz, CA, diluted 1:1500) was added to the membrane and incubated for 2 h. Then, the membrane was washed 3 times with PBST, and a horseradish peroxidase-conjugated goat anti-rabbit secondary antibody (Sigma, diluted 1:5000) was added to the membrane and incubated for 1 h. The membrane was washed 4 times with PBST. An electrochemiluminescence (ECL) colouring system (Amersham Life Sciences) was used to develop the blots.

### Apolipoprotein A5 measurement

An enzyme-linked immunosorbent assay (ELISA) was used for serum ApoA5 detection with a commercial ELISA kit (LSBio).

### Statistical analysis

The SPSS 16.0 statistical software package was used for the data analysis. The data are presented as the mean ± standard deviation (x ± s). The *t* test was used to compare the serum ApoA5 levels between the control group and the HBV patient group. A *p* value <0.05 was considered statistically significant.

## Results

### Subjects

Baseline characteristics of the participants are shown in Table 1. There were no significant differences in gender, age and BMI between the HBV patient and healthy individuals (P > 0.05), and significant differences were found between the two groups in terms of ALT and AST (*P* < 0.001).

**Table 1:** Clinical and biochemical characteristics of the subjects enrolled in the present study

Clinical parameters	Healthy individuals (*N* = 125)	HBV patients (*N* = 221)	*P*-value
Age (years)	48.7 ± 13.6	51.6 ± 11.8	0.126
Sex (male/female)	75/50	120/101	0.247
BMI	25.4 ± 1.8	24.9 ± 1.2	0.352
HBeAg (+/−)	NS	47/174	ND
HBV DNA (copies/ml)	<500	5.8E + 06 ± 4.2E + 08	ND
ALT (IU/l)	<30	193.4 ± 187.5	<0.001
AST (IU/l)	<30	185.8 ± 170.2	<0.001

A significant at *p* value ≤ 0.05

*N* number of the subjects, *NS* none sense, *ND* not done, *BMI* body mass index, *ALT* alkanine aminotransferase, *AST* aspartate aminotransferase

### ApoA5 mRNA and protein expression was decreased in HepG2.2.15 cells

**Fig. 1:**
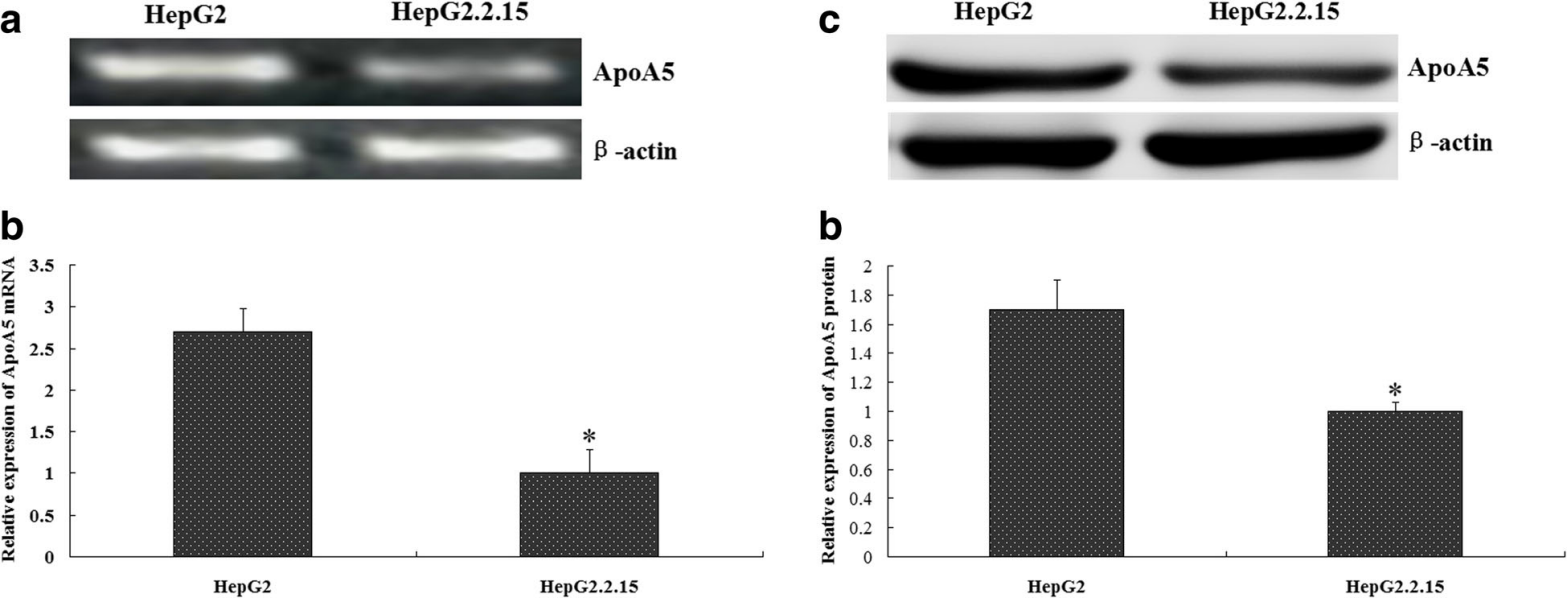
Comparison of ApoA5 mRNA and protein expression in HepG2 cells and HepG2.2.15 cells. **a** and **b** The relative levels of ApoA5 mRNA between HepG2 and HepG2.2.15 cells were determined by RT-PCR. **c** and **d** ApoA5 protein expression in HepG2 and HepG2.2.15 cells was determined by western blotting

HepG2.2.15 cells are HepG2 cells that stably transfected with the HBV genome, and can express viral RNA and protein, synthetise and secrete the complete virus-like particles [7]. Using RT-PCR and western blotting, we examined ApoA5 expression in the HepG2 and HepG2.2.15 cells. As shown in Fig. 1, the ApoA5 mRNA and protein expression in the HepG2.2.15 cells were significantly reduced compared with those in the HepG2 cells.

### The serum ApoA5 levels were decreased in HBV patients

**Fig. 2:**
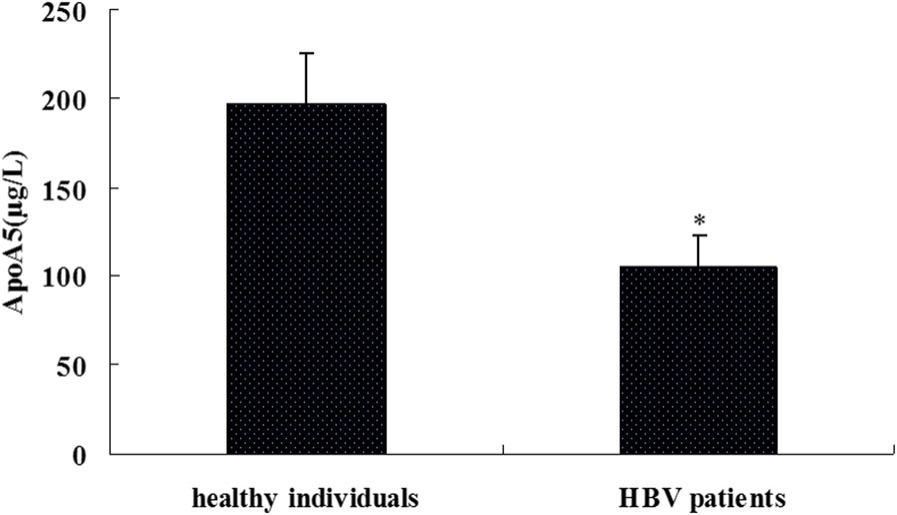
Comparison of serum ApoA5 levels between the healthy individuals and HBV patients. The serum ApoA5 levels in 125 healthy individuals and 221 HBV patients were measured by ELISA. *P < 0.005

The serum ApoA5 levels in the HBV patients and healthy individuals were measured by ELISA. The results showed that the ApoA5 concentration was 196.4 ± 28.7 μg/L in the healthy individuals and 104.5 ± 18.3 μg/L in the HBV patients. The statistical analysis results showed that the ApoA5 levels were significantly decreased in the HBV patient group compared with that in the healthy control group (*P* < 0.05, Fig. 2).

### HBV inhibited ApoA5 promoter activity, mRNA and protein expression

**Fig. 3:**
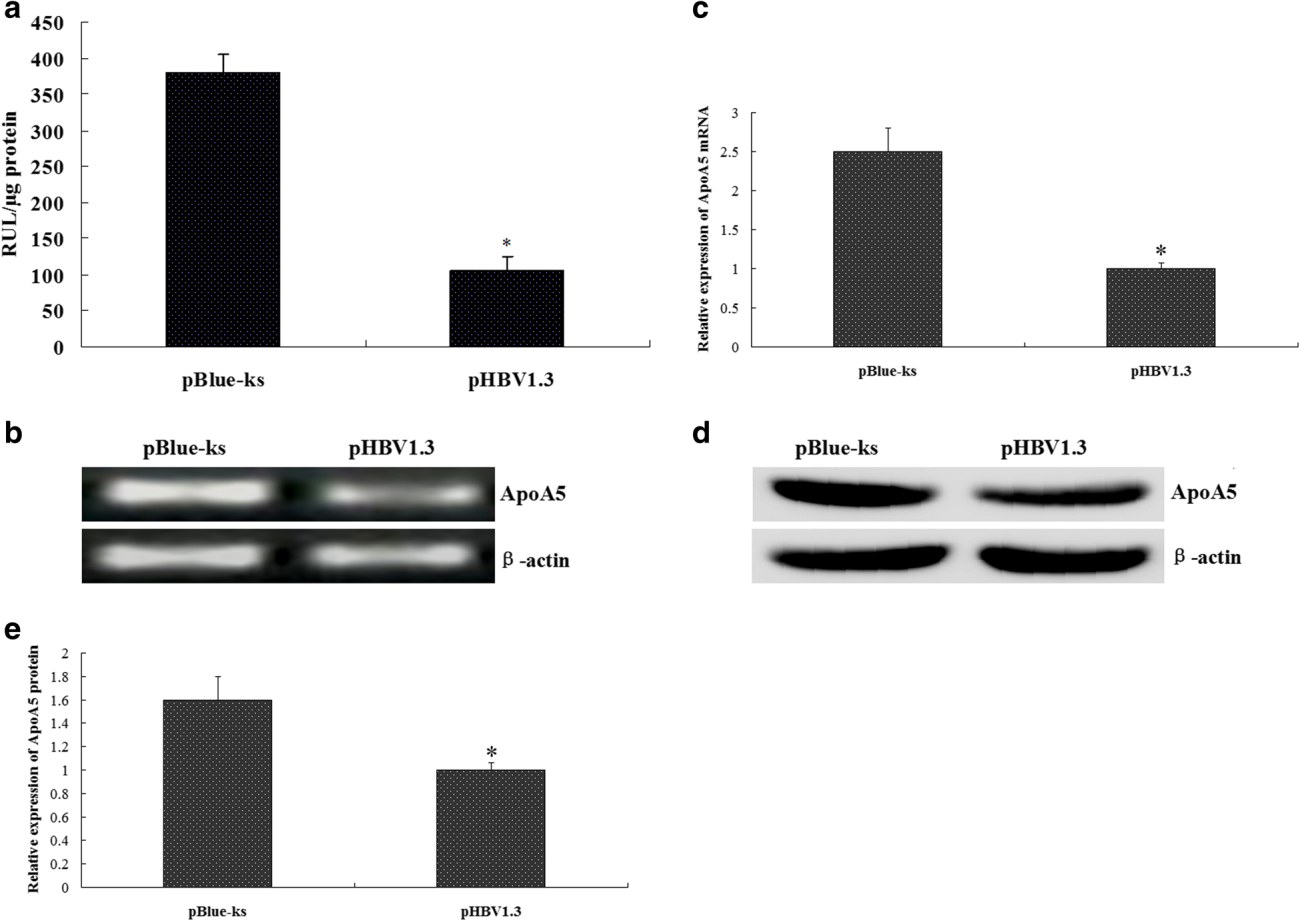
HBV inhibited ApoA5 promoter activity, mRNA and protein expression. **a** Effect of HBV on ApoA5 promoter activity, *P < 0.005. HepG2 cells were cotransfected with pHBV1.3 and the ApoA5 gene promoter pGL-ApoA5-LUC plasmid, and transfection with pBlue-ks was used as a control, the luciferase activity was measured at 48 h post-transfection. **b** and **c** Effects of HBV on ApoA5 mRNA expression. HepG2 cells were transfected with pHBV1.3 or pBlue-ks, 48 h after transfection, ApoA5 mRNA was determined by RT-PCR. **d** and **e** Effects of HBV on ApoA5 protein expression. HepG2 cells were transfected with pHBV1.3 or pBlue-ks, 48 h after transfection, ApoA5 protein was determined by western blotting

To investigate the molecular mechanism underlying the inhibition of ApoA5 expression by HBV, we cotransfected HepG2 cells with HBV infectious clone pHBV1.3 and the ApoA5 gene promoter pGL-ApoA5-LUC plasmid. Transfection with the empty vector pBlue-ks was used as a control [8]. The efficiency of transfection was estimated by the number of green fluorescence-emitting, 24–48 h after transfection, the total number of green fluorescent cells revealed 55–65 % transfection efficiency. The regulation of the ApoA5 promoter by HBV was determined by measuring the luciferase activity. As shown in Fig. 3a, the luciferase activity was significantly reduced (105.6 ± 18.7 RUL/μg protein) after transfection with pHBV1.3 compared with pBlue-ks transfection (379.5 ± 26.4 RUL/μg protein). We further examined the changes in ApoA5 mRNA and protein expression. The results showed that the ApoA5 mRNA and protein expression levels were decreased after pHBV1.3 transfection compared with pBlue-ks transfection (Fig. 3b, c, d and e).

### HBV inhibited ApoA5 expression through its core gene

**Fig. 4:**
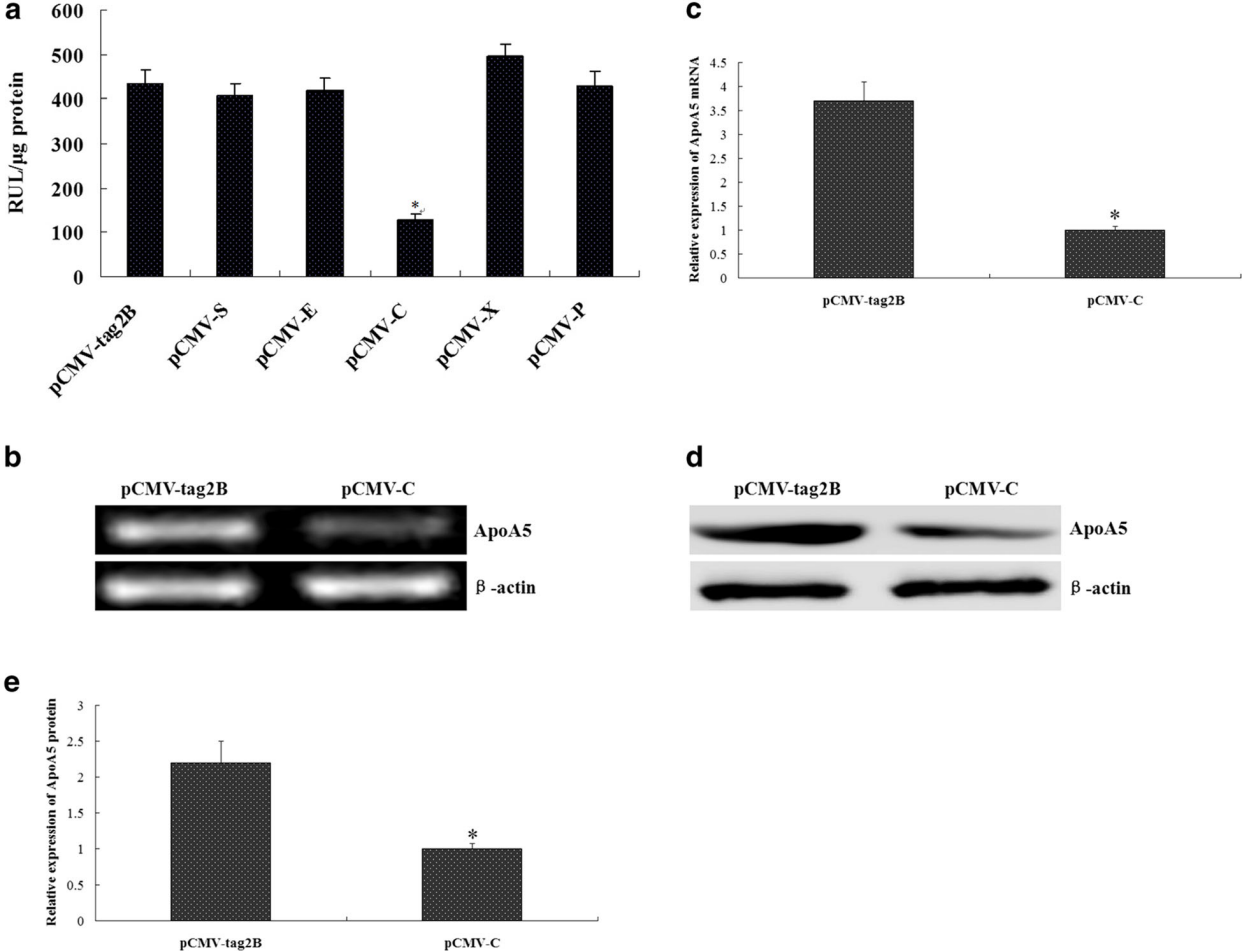
HBV inhibited ApoA5 expression through its core gene. **a** Effect of HBV core gene on ApoA5 promoter activity, *P < 0.005. HepG2 cells were transfected with pCMV-S, pCMV-E, pCMV-C, pCMV-X, pCMV-P and pCMV-tag2B, the luciferase activity was measured at 48 h post-transfection. **b** and **c** Effects of HBV core gene on ApoA5 mRNA expression. HepG2 cells were transfected with pCMV-C or pCMV-tag2B, 48 h after transfection, ApoA5 mRNA was determined by RT-PCR. **d** and **e** Effects of HBV core gene on ApoA5 protein expression. HepG2 cells were transfected with pCMV-C or pCMV-tag2B, 48 h after transfection, ApoA5 protein was determined by western blotting

Eukaryotic expression plasmids containing all of the HBV genes (pCMV-S, pCMV-E, pCMV-C, pCMV-X, and pCMV-P) were cotransfected into HepG2 cells with the ApoA5 gene promoter pGL3-ApoA5-LUC plasmid. pCMV-tag2B was used as an empty control. 24–48 h after transfected with pIRES2-EGFP, the transfection efficiency is 55–65 %. The regulatory effect of the proteins on the ApoA5 gene promoter was examined by measuring the luciferase activity. The results showed that the HBV core gene had a significant inhibitory effect on the ApoA5 gene promoter, whereas the other HBV genes did not have a significant regulatory effect (Fig. 4a). Finally, we examined the changes in ApoA5 mRNA and protein expression. The results showed that ApoA5 mRNA and protein expression was decreased after transfection with the HBV core gene compared with that after transfection with pCMV-tag2B (Fig. 4b, c, d and e), suggesting that HBV might inhibit ApoA5 expression through its core gene.

## Discussion

In the present study, we investigated the differences in ApoA5 expression in HepG2.2.15 and HepG2 cells. We also examined the serum ApoA5 levels in HBV patients and healthy controls. We found that HBV inhibited ApoA5 expression both in vivo and in vitro. To investigate the molecular mechanism by which HBV regulated ApoA5 expression, we used the luciferase reporter gene system to examine the regulatory effect of the infective HBV clone pHBV1.3 on the ApoA5 gene promoter and found that pHBV1.3 inhibited ApoA5 gene promoter activity. pHBV1.3 also inhibited ApoA5 expression at both the transcriptional and translational levels. Additionally, we cotransfected all of the HBV genes with the ApoA5 gene promoter pGL3-ApoA5-LUC plasmid into HepG2 cells. By measuring the luciferase activity, we found that the HBV core gene inhibited ApoA5 gene promoter activity. The RT-PCT and western blottingassays confirmed that the core gene inhibited ApoA5 expression at the mRNA and protein levels. Therefore, HBV may inhibit ApoA5 expression through its core gene.

The liver is involved in the synthesis of almost all lipoproteins and the enzymes and proteins involved in lipoprotein metabolism [9]. The liver is also the major site of lipoprotein degradation and plays a very important role in lipid metabolism [10, 11]. Studies have confirmed that many viral infections change the blood lipid metabolism [5, 12–14]. For example, serum high-density lipoprotein (HDL), low-density lipoprotein (LDL), total cholesterol (TC) and apolipoprotein B (ApoB) levels are decreased in hepatitis C virus-infected patients [15–17]. Our previous studies also found that HBV inhibited the synthesis and secretion of ApoB by inhibiting microsomal triglyceride transfer protein (MTP) expression [18], and the levels of serum ApoM was significantly elevated in patients as compared to healthy individuals, and enhanced ApoM levels in HBV infection may in turn suppress HBV replication [19]. Moreover, both Wang et al. and Jiang et al. confirmed that HBV inhibited the synthesis and secretion of ApoA1 in vivo and in vitro [20, 21].

ApoA5 has a stronger surface repulsion force and lipid binding ability. Its concentration in plasma is very low, but it plays an important role in in vivo blood lipid metabolism. ApoA5 plays a role in elevating HDL levels and is closely associated with hypertriglyceridemia [22, 23]. In the present study, we found that the serum ApoA5 content was decreased in HBV patients. Jiang et al. examined the serum HDL-C content in HBV patients and healthy controls and found that HDL-C was decreased in HBV patients [20]. Because ApoA5 plays a role in elevating HDL-C, the decrease in the serum HDL-C content in HBV patients may be related to the inhibition of ApoA5 expression by HBV.

Several researches have demonstrated a protective role of HBV/HCV infection in the progression of atherosclerosis because of the influence of the viruses on lipid metabolism [24, 25] Our results are consistent with the conclusion, which may shed new light on the effect of HBV infection in the progression of atherosclerosis.

## Conclusion

In summary, in the present study, we demonstrated that HBV inhibited ApoA5 synthesis and secretion based on both in vivo and in vitro experiments. However, elucidating the detailed molecular mechanism of how HBV core gene inhibits the activity of ApoA5 gene promoter requires further investigation.

### Limitations and future study

The retrospective nature of this study is a limitation. Firstly, we had no knowledge on the habitual diet of the participants, which could influence lipids levels. However, it seems unlikely that the HBV patients would have had a diet that differed substantially from that of the healthy individuals. Secondly, the role of anti-HBV drugs on the serum ApoA5 levels were not considered. Thirdly, it is difficult to avoid a data bias from the analysis because of derandomization and the sample size. Therefore, large scale studies are needed for future prospective research to determine the influence of the factors mentioned above on the serum ApoA5 levels.
